# Controlling Hypertension After Severe Cerebrovascular Event (CHASE): study protocol for a randomized controlled trial

**DOI:** 10.1186/s13063-018-2530-x

**Published:** 2018-02-21

**Authors:** Fang Yuan, Fang Yang, Changhu Xue, Kangjun Wang, Qiuwu Liu, Jun Zhou, Feng Fu, Xiaocheng Wang, Wei Zhang, Yi Liu, Kang Huo, Hua Lv, Wen Jiang, on behalf of the CHASE Study Group

**Affiliations:** 1 Department of Neurology, Xijing Hospital, Fourth Military Medical University, Xi’an, 710032 China; 2 Department of Neurology, Xianyang Central Hospital, Xianyang, 712000 China; 3 Department of Neurology, Hanzhong Central Hospital, Hanzhong, 723000 China; 4 Department of Neurology, Xi’an 141 Hospital, Xi’an, 710000 China; 5 Department of Neurology, Shangluo Central Hospital, Shangluo, 726000 China; 6 Department of Neurology, 215 Hospital of Shaanxi NI, Xianyang, 712021 China; 7 Department of Neurology, Yulin No.2 Hospital, Yulin, 719000 China; 8 Department of Neurology, Tangdu Hospital, Fourth Military Medical University, Xi’an, 710038 China; 9 Department of Neurology, Ankang Central Hospital, Ankang, 725000 China; 10 Department of Neurology, The First Affiliated Hospital of Xi’an Jiaotong University, Xi’an, 710061 China; 11 Department of Neurology, Shaanxi Provincial People’s Hospital, Xi’an, 710068 China; 12 The Shaanxi Cerebrovascular Disease Clinical Research Center, Xi’an, 710032 China

**Keywords:** Blood pressure, Severe stroke, Hypertension, Prognosis, Critical care

## Abstract

**Background:**

No ideal blood pressure (BP) range has been scientifically determined for acute stroke, and no studies on BP management have been carried out for patients with severe stroke. This trial aims to investigate whether individualized lowering of elevated BP would improve the outcome in patients with severe stroke.

**Methods/design:**

The CHASE trial is a multicenter, randomized, controlled study. A total of 500 adult patients with acute severe stroke will be enrolled in 18 study sites in China and randomized to individualized BP lowering (10–15% reduction from admission level) or guideline-recommended BP lowering. The primary outcome measurement is the proportion of participants with a poor outcome (modified Rankin Scale ≥ 3) at day 90 of enrollment. Secondary outcomes include disability at hospital discharge and the ability of activities of daily living at day 90 of enrollment. The relationship between intervention and the primary outcome will be analyzed using multivariate logistic regression adjusted for study site, demographics, and baseline characteristics.

**Discussion:**

The CHASE trial will be the first study to explore the optimum BP management for acute severe stroke. This trial potentially offers a strong argument for individualized target for lowering elevated BP in patients with severe stroke.

**Trial registration:**

ClinicalTrials.gov, NCT02982655. Registered on 30 November 2016.

**Electronic supplementary material:**

The online version of this article (10.1186/s13063-018-2530-x) contains supplementary material, which is available to authorized users.

## Background

Elevated blood pressure (BP) is one of the most common clinical manifestations in acute stroke; 70–84% [1–3] of stroke patients have a systolic blood pressure (SBP) > 140 mmHg on admission. Despite efforts by a few multicenter studies, the optimum management of BP in acute stroke is still an ongoing dilemma. The ACCESS study and CHHIPS study indicated that modest BP reduction brought down the mortality and the number of vascular events [4], whereas the SCAST study suggested that a careful BP-lowering treatment was associated with a higher risk of poor outcome. The results from the COSSACS study, CATIS study, ATACH-2 study, and INTERACT2 study are neutral: intensive lowering of BP did not reduce the mortality or severe disability significantly [5–8].

However, none of the above studies were targeted at patients with severe stroke. Acute severe strokes account for 13–24% of all strokes [9, 10] and are associated with cerebral hernia, acute cardiac failure [11], systemic complications [12], and death. Given the paucity, we initiated a multicenter randomized controlled trial (RCT) called CHASE, dedicated to test whether individualized antihypertensive treatment would lower the mortality and major disability in patients with severe stroke.

## Methods

### Study objectives

The study aims at assessing the effect of individualized BP-lowering regimens vs guideline-recommended BP-lowering regimens on mortality and morbidity in patients with acute severe stroke.

### Study design

**Fig. 1:**
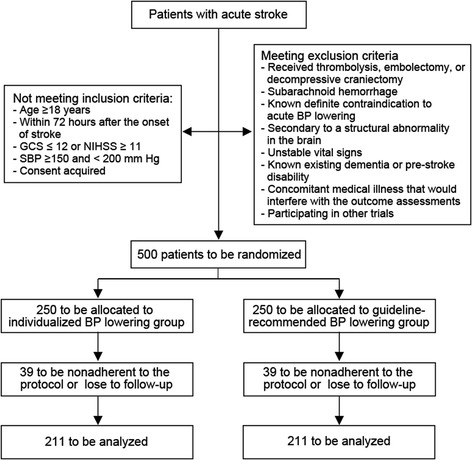
*Flowchart* of participants in the CHASE trial. BP blood pressure, GCS Glasgow Coma Scale, NIHSS National Institute of Health stroke scale, SBP systolic blood pressure

This study has a multicenter, randomized, controlled, single-blind design. Patients will be randomized to two different arms: (1) intervention group (individualized BP lowering), with an individualized target for BP lowering during the first week of hospitalization; and (2) control group (guideline-recommended BP lowering), with a fixed target recommended by the guideline for BP lowering during the first week of hospitalization. The participants are blind to the grouping results; the researchers are informed of the grouping results because they have to follow a specified strategy of BP management. Figure 1 shows a flowchart of the study design. Recommendations for Interventional Trials (SPIRIT) Figure are outlined in Additional file 1.

### Study sites

This is a Chinese multicenter trial involving 18 tertiary and district general hospitals in Shaanxi province, an administrative region with a population of 38 million located at the very center of China. The study will be conducted according to Good Clinical Practice guidelines and the Declaration of Helsinki. The study has been approved by the ethics committees of Xijing hospital (KY20162085-2).

### Training of investigators

All CHASE investigators are required to be trained in the protocol, Good Clinical Practice, and use of the Glasgow Coma Scale (GCS), National Institute of Health stroke scale (NIHSS), Barthel index, and modified Rankin Scale (mRS) if they had no recent certifications.

### Study population

The study will include patients with acute severe stroke who have elevated BP on admission. In order to be eligible for inclusion in the study, the patients have to comply with the following criteria: (1) age ≥ 18 years; (2) the randomly assigned BP-lowering regimen is able to be commenced within 72 h after the onset of stroke (ischemic or hemorrhagic), confirmed by a computed tomography (CT) or magnetic resonance imaging (MRI) scan of the brain (if the precise timing of the onset of symptoms or signs of the qualifying event is unknown, then the time of onset will be taken as the last time the patient was known to be well); (3) GCS on admission ≤ 12 or NIHSS on admission ≥ 11; (4) there are at least two SBP measurements of ≥ 150 and ≤ 210 mmHg, recorded ≥ 5 min apart (patients with an initial SBP < 150 mmHg may be randomized when the SBP fulfils entry criteria on rechecking up to 72 h after the onset of stroke); and (5) written informed consent is able to be obtained directly from the patient or an appropriate surrogate, based on local ethics committee recommendations.

Exclusion criteria are: (1) patients who have received thrombolytic therapy, embolectomy, or decompressive craniectomy for the current stroke; (2) patients with subarachnoid hemorrhage; (3) known definite contraindication to acute BP lowering (e.g. known severe carotid, vertebral, or cerebral arterial stenosis, Moyamoya disease or Takayasu’s arteritis, high grade stenotic valvular heart disease); (4) secondary to a structural abnormality in the brain (e.g. an arteriovenous malformation, intracranial aneurysm, tumor, or trauma); (5) unstable vital signs and requiring the use of vasoactive agents; (6) known existing dementia or pre-stroke disability (e.g. score 3–5 on the modified Rankin scale); (7) concomitant medical illness that would interfere with the outcome assessments and/or follow-up (advanced cancer; severe pulmonary dysfunction [forced expiratory volume in 1 s < 50%]; severe cardiac dysfunction [ejection fraction ≤ 50%]; severe hepatic failure [Child-Pugh score ≥ 7]; severe renal failure [glomerular filtration rate ≤ 30 mL/min or serum creatinine ≥ 4 mg/dL]); (8) patients who are currently participating in other investigational trials; and (9) patients who are considered to have a high likelihood of not adhering to the study treatment or the follow-up regimen.

### Randomization

A secure website (http://traillogin.applinzi.com) will be used to perform the centralized randomization (computerized random numbers). Patients will be randomized into one of the two intervention arms (1:1).

### Trial interventions

In patients who are assigned to receive individualized regimen to lower BP (individualized BP-lowering group), antihypertensive treatments are to be initiated to reduce SBP to a range of 130–180 mmHg and by 10–15% from the admission level within 2 h after randomization. SBP in the individualized BP-lowering group is to be maintained around the target level for one week with or without the use of hypertensive agents. In participants who were assigned to receive guideline-recommended treatment [13, 14] (guideline-recommended BP-lowering group), antihypertensive agents are to be administered if the SBP was > 200 mmHg in acute ischemic stroke (AIS) or the SBP was > 180 mmHg in intracerebral hemorrhage (ICH). The goal is to maintain the SBP < 200 mmHg in ischemic stroke and < 180 mmHg in ICH for one week with or without the use of hypertensive agents. Table 1 presents the detailed management of BP in the first week after randomization.

**Table 1:** Management of BP during the first week after randomization

SBP level	Approaches
*Individualized BP-lowering group*	
Above the range^a^	Increase the dose of AHD or use other stronger AHDs
In the range^a^	Maintain the regimen
Below the range^a^ and > 100 mmHg	Reduce the dose of AHD or withdraw AHD
< 100 mmHg	Use vasopressor agents
*Guideline-recommended BP lowering-group*	
> 200 mmHg in AIS > 180 mmHg in ICH	Increase the dose of AHD or use other stronger AHDs
< 200 and > 100 mmHg in AIS < 180 and > 100 mmHg in ICH	Use the least dose of AHD to keep SBP not > 200 mmHg in AIS, 180 mmHg in ICH
< 100 mmHg	Use vasopressor agents

^a^10–15% reduction from admission level and in the range of 130–180 mmHg

*AHD* antihypertensive drug, *AIS* Acute ischemic stroke, *BP* blood pressure, *ICH* intracerebral hemorrhage, *SBP* systolic blood pressure

The selection of antihypertensive agents is based on the local availability; no specific agent is stipulated (both intravenous and oral agents can be used). Patients who take antihypertensive agents before stroke continue to take the same antihypertensive medication (same drug with same dosage). The rest of the medical care for stroke is based on the guideline [15, 16]. The management of BP after the first week of hospitalization adheres to the guideline as well.

### Study procedures

Table 2 presents all the variables measured at each time point of the study. At the time point of baseline screening, demographics, subtypes of stroke, medical history (e.g. AIS, ICH, coronary event, diabetes mellitus, and hypertension), physical examination, clinical scores (NIHSS, GCS, Barthel index, mRS), and vital signs are recorded. On day 1 and day 7, routine laboratory tests for stroke patients are assessed, including blood routine, liver and renal function test, serum lipid, fasting glucose, urine routine, and electrocardiography. Co-morbidities on the day of screening and hospital discharge are recorded. Clinical scores on day 7, the day of hospital discharge, and day 90 are collected. During the whole hospitalization, BP, vital signs, and adverse events (AEs) are monitored, and concomitant treatments are documented.

**Table 2:** Timing and content of study assessments

Items	Day of enrollment									
	Screening	1	2	3	4	5	6	7	HD	90
Written informed consent	●									
Inclusion and exclusion criteria	●									
Demographics	●									
Medical history	●									
Physical examination	●							●	●	
BP monitoring	●	●	●	●	●	●	●	●	●	
Laboratory tests		●						●		
NIHSS and GCS	●								●	
Barthel index and mRS	●								●	●
Vital signs monitoring	●	●	●	●	●	●	●	●	●	
Co-morbidities	●								●	
AEs		●	●	●	●	●	●	●	●	
Use of antihypertensive agents		●	●	●	●	●	●	●	●	
Concomitant therapies		●	●	●	●	●	●	●	●	

*HD* hospital discharge, *BP* blood pressure, *GCS* Glasgow Coma Scale, *NIHSS* National Institute of Health stroke scale, *mRS* modified Rankin Scale, *AE* adverse event

### Blood pressure monitoring

An automatic BP cuff on the unaffected arm (right arm is chosen when both sides are affected or unaffected) is used to monitor the BP (SBP, diastolic BP, and mean arterial pressure). During the first 24 h after randomization, BP is recorded every 2 h. On days 2 and 3, BP is recorded every 4 h. During days 4–7, BP is recorded every 8 h. On the day of hospital discharge, BP at 08:00 is recorded. Three measurements of BP are required for each time point and the average is recorded.

### Data quality

The following procedures to minimize bias and ensure data quality and protocol standardization are required: (1) a training session will be held for research coordinators from all the participating centers before the commencement of CHASE study; (2) the principal investigator in each research site takes charge of quality control by supervising the conduct of the trial in accordance with the prespecified protocol, applicable guidelines, and regulations; (3) all participating sites will have monitoring visits via video conferencing after every ten patients are randomized, to verify consent, eligibility criteria, anomalous data, and reported serious AEs; (4) the Shaanxi Cerebrovascular Disease Clinical Research Center (SCRC) will prepare monthly reports on recruitment, randomization, data completeness, and protocol adherence. Case report forms and informed consent forms will be securely stored in a locked cabinet in a secure office with limited access. All digital data will be password-protected and stored in a firewall-protected secure environment. The trial sponsor has access to the final trial dataset.

### Outcome measurement

The primary outcome measurement is the proportion of participants with a poor outcome at day 90 of enrollment. Poor outcome is defined as major disability (mRS ≥ 3, unable to live independently) or all-cause death. The proportion of participants with a poor outcome at hospital discharge is the key secondary outcome. Other secondary outcomes are the disability (evaluated by NIHSS, GCS, and Barthel index) at hospital discharge and the ability of activities of daily living at day 90 of enrollment evaluated by Barthel index. Outcomes are assessed independently by trained research assistants who are blind to the grouping results and clinical data. The 90-day outcomes are evaluated via telephone interviews at day 90 of enrollment (a delay of up to three days is acceptable). In cases when the patient is incapable to complete the interview, the first choice for a proxy is the spouse/live-in companion.

### Determination of the sample size

The sample size was set at 500 to provide at least 80% power to detect a 6% absolute risk reduction in the primary outcome for patients in the individualized BP-lowering group compared to those in the guideline-recommended control group, using a two-sided significance test with 5% type I error. The following assumptions were made: a primary outcome of 60% in the control group will be reduced to 54% in the individualized group; and there will be 10% non-adherence to the treatment protocol and 5% overall loss to follow-up. The 60% incidence of unfavorable 90-day outcome is extrapolated from one Chinese study on severe stroke (NIHSS > 10) in 2013 [17]. The 10% non-adherence rate and the absolute risk reduction in primary outcome are extrapolated from the INTERACT1&2 studies which were designed to compare the effects of two different BP-lowering strategies in acute stroke patients [8, 18].

### Statistical and analytical plan

Patients will be analyzed according to the intention-to-treat principle. Analyses will be conducted at the Shaanxi Cerebrovascular Disease Clinical Research Center by blinded biostatisticians. Baseline characteristics will be summarized using univariate analyses. Categorical variables will be presented as the number and percent, whereas the continuous variables will be presented as mean (± standard deviation) or median (interquartile range). We will compare the proportions for the primary and secondary outcomes between patients randomized to the individualized BP-lowering group vs the guideline-recommended BP-lowering group using the Chi-square test. We will calculate the relative risk reduction, absolute risk reduction, and the number needed to treat with individualized BP-lowering target to prevent one death. Logistic multivariate analyses will be used to adjust for potential confounding effects of different variables and estimate adjusted odds ratios and associated 95% confidential intervals. Two-sided *p* values ≤ 0.05 will be considered significant. Statistical analysis will be performed with SPSS version 22 software (SPSS Inc., Chicago, IL, USA).

### Protocol amendments

Protocol amendments will be agreed upon with the CHASE Study Group, Sponsor and Funding Body before submission for ethical approval. Following ethical approval, protocol modifications will be communicated with relevant parties such as the trial investigators, the trial registry, and, if required, trial participants.

### Dissemination policy

The results of this trial will be disseminated to a wide clinical audience (patients, health professionals, policy makers, and the general public) through publication in a high-impact international scientific journal.

## Discussion

A few multicenter, randomized studies have been carried out to search for the ideal BP range for patients with acute stroke; however, none of them aimed at severe stroke, which has a much higher rate of mortality and disability. No clinical evidence can be used to guide the BP management in critical care for those stroke patients with an initial presentation of severe neurological deficits. Our study aims to test the superiority of individualized BP lowering compared to guideline-recommended BP lowering in improving three-month outcome. Our study will provide the first evidence on the optimum BP-lowering target for patients with acute severe stroke.

Factors that contribute to raised BP in acute stroke are multifaceted, such as history of hypertension, stroke severity, pressure response to hospital admission, infarct area related to BP adjustment, and increased intracranial pressure [19]. Raised BP later falls into the following types: without antihypertensive medications BP declines spontaneously; with antihypertensive medications BP does not decline, declines modestly (10–15% from baseline), or intensively (≥ 20% from baseline value) [20]. Elevated BP raises the risk of cerebral edema, hemorrhagic transformation of the infarct [21], hematoma expansion [22], cardiac complications, and renal insufficiency. On the other hand, a decrease in BP might reduce the blood perfusion of the brain and aggravate the cerebral ischemic injury, especially in large territorial ischemia [23]. One clinical study indicated that both large increase and reduction in BP (> 20 mmHg) were associated with a higher risk of early neurological deterioration, increased infarct volume, and poorer outcome at three months [24]. Therefore, a proper management of BP is essential for stroke patients.

However, no ideal BP range has been scientifically determined yet. Guidelines for AIS from the American Heart Association (AHA)/American Stroke Association suggest that BP targets are based on best clinical judgment and a reasonable estimate might be to lower the SBP by 15% if the BP is > 220/120 mmHg [15]. For ICH patients presenting with SBP < 180 mmHg, guidelines from AHA in 1999 suggest to defer antihypertensive therapy; guidelines from AHA in 2015 suggest to lower SBP to 140 mmHg. Chinese guidelines for AIS recommend the use of hypertensive therapy when SBP is > 200 mmHg [14]. Also, Chinese guidelines for ICH suggest that antihypertensive therapy should be initiated when SBP is > 180 mmHg and a target around 160/90 mmHg can be considered although evidence is lacking [25]. None of the above guidelines mentioned any BP-lowering regimen specifically for severe stroke due to the lack of clinical evidence.

Given the paucity of studies on BP management for severe stroke, we performed a retrospective preliminary study in stroke patients admitted to neurological intensive care unit (NICU) in Xijing Hospital (a tertiary teaching hospital) in the last three years and found that NICU mortality was significantly higher in patients with SBP reduced by > 15% than those with SBP reduced by < 15%. This difference of mortality remained significant in patients with AIS. For ICH, the NICU mortality was also higher in patients with SBP reduced by > 15% than those with SBP reduced by < 15%, albeit insignificant.

Based on the above result and previous studies, we designed a multicenter RCT to investigate whether individualized lowering (10–15% reduction from admission level) of elevated BP would improve the outcomes in patients with severe stroke, hoping to provide evidence of BP management for acute severe stroke, help improve the quality of life, and reduce family and social burdens.

### Trial status

The CHASE study trial was conceived and designed in 2016. At the time this manuscript was submitted, full approval by the Medical Ethics Committee has been obtained for all centers and recruitment was started. The patients’ follow-up is currently ongoing.

## Additional file

Additional file 1: SPIRIT 2013 checklist: recommended items to address in a clinical trial protocol and related documents. (PDF 115 kb)

## Abbreviations

AHA: American Heart Association
AIS: Acute ischemic stroke
BP: Blood pressure
GCS: Glasgow Coma Scale
ICH: Intracerebral hemorrhage
NIHSS: National Institute of Health stroke scale
SBP: Systolic blood pressure
